# Letters to the editor: Association between Job Strain and Nutrient Intake by Diet in Working Population: the Role of Shift Work.

**DOI:** 10.2188/jea.16.220

**Published:** 2006-09-04

**Authors:** Gloria Marchetti, Luca Coppeta, Maria Rosa Bollea, Antonio Pietroiusti, Andrea Magrini, Norito Kawakami, Akizumi Tsutsumi, Takashi Haratani, Fumio Kobayashi, Masao Ishizaki, Takeshi Hayashi, Osamu Fujita, Yoshiharu Aizawa, Shogo Miyazaki, Hisanori Hiro, Takeshi Masumoto, Shuji Hashimoto, Shunichi Araki

**Affiliations:** 1 Clinical Nutrition Department. University of Rome; 2 Occupational Medicine Department. University of Rome; 3 Department of Mental Health, University of Tokyo Graduate School of Medicine; 4 University of Occupational and Environmental Health; 5 Japan National Institute of Occupational Safety and Health; 6 Aichi Medical University; 7 Kanazawa Medical University; 8 HITACHI Information & Telecommunication Systems Shinkawasaki Health Care Center; 9 Kariya General Hospital Eastern Division; 10 Kitasato University School of Medicine; 11 Meiji University Law School; 12 Adecco Health Support Center; 13 Mitsubishi Chemical Corporation; 14 Fujita Health University School of Medicine; 15 National Institute of Occupational Safety and Health, Japan

Dear Sir,

A paper recently published by Kawakami et al^1^ analyzed the association between job strain, worksite support and nutrient intake among a large working population in Japan, in order to evaluate whether work-related dietary habits may represent the factor underlying the association between job stress and cardiovascular disease reported in previous studies.

They assessed the degree of psychological stress using the Karasek's Job Content Questionnaire and evaluated nutritional intake of several macro and micronutrients by a 31-item dietary history questionnaire. They analyzed the relationship between job stress and nutrient intake separately in men and women after controlling for many potential confounders, and found a significant association, only in males, between high work strain, total fat intake, and both mono- and polyunsaturated fatty acids.

The findings are interesting; however we have some criticisms. First of all, data on weight and height of participants are not given. Differences in these parameters may influence both energy expenditure (i.e. Harris-Benedict formulae) and basal metabolic requests. Therefore, the intake of nutrients may in turn be related to basal differences in the above mentioned parameters. Furthermore, many studies have demonstrated the possible influence of different job strains on body mass index distribution among working population. Thus, this parameter (body mass index) should be separately evaluated, in the statistical analysis as a possible confounding factor.

Although not explicitly told out in the work, it seems reasonable to suppose that the working population was largely made by shift and night workers, because it included a substantial fraction of factory employers. Many studies have shown that shift work is associated with both job strain and dietary nutrient intake.^2^^,^^3^ Rotating shift may play an important role in the change of dietary habits of employers by the interference with biological rhythms and recreational activities of working populations. It has been also demonstrated that shift work can be associated with the prevalence and the severity of other important cardiovascular risk factor such as increased blood pressure, lipid and carbohydrate metabolism disturbances, and also metabolic syndrome.^4^^,^^5^^,^^6^ The preliminary data of a work that we are carrying out in a large working population (over 900 employed men and women) show a consistent association between shift work, dietary changes and high total fat intake.

Therefore, we think that it could be useful to evaluate the prevalence of shift workers and night workers in the four degrees of job strain categories, and that the total and specific nutrients intake should be separately evaluated in shift workers and daytime workers.

Finally, we think that the large dataset that Kawakami et al have collected in their population might be improved by considering other important working conditions (i.e. sedentariness) in determining the dietary habits of working populations and the possible correlation of nutritional intake with an increased risk of cardiovascular disease or other disorders.

Gloria Marchetti (Clinical Nutrition Department. University of Rome), Luca Coppeta (Occupational Medicine Department. University of Rome), Maria Rosa Bollea (Clinical Nutrition Department. University of Rome), Antonio Pietroiusti, (Occupational Medicine Department. University of Rome), Andrea Magrini (Occupational Medicine Department. University of Rome).

Address for corresponding: Luca Coppeta, Occupational Medicine Department, University of Rome, "Tor Vergata", Via di Torregaia 122, 00133 Rome, Italy. (e-mail: lcoppeta@tiscali.it)

## Reply

Dear Sir,

We thank Coppeta et al^1^ for responding to our article.^2^ We agree that all the issues pointed out are very relevant and important. First, data on body mass index (BMI) were available for part of the sample (14,197 men and 2,694 women). Controlling for BMI does not change the main findings. There was no statistically significant difference in BMI among the groups classified on the basis of job strain, job demands, job control, or worksite support, after controlling for the covariates (p>0.05). In our study, for both men and women, total energy intake was greater for the groups with higher worksite support.^2^ The greater total energy intake may be compensated for by means of possible greater physical activity among groups with higher worksite support. The association between job strain and waist-to-hip ratio based on part of the sample was published elsewhere.^3^ We do not have information on other indicators of body composition for the whole sample.

We agree on the importance of shift work as a determinant of job stressors and nutritional intakes. The prevalence of shift work was not so high in our sample: A total of 17% of men and 9% of women engaged in rotating shift work. We had conducted analyses controlling for rotating shift work, in which the main findings were unchanged. While the association of shift work with job strain and nutritional intakes is also of interest, we do not go into the detail here because a major focus of our article^2^ was on the association between job strain and nutritional intakes.

There are several behavioral risk factors for coronary heart disease other than nutritional intakes, including sedentary work and smoking. Inter-correlations and accumulation/clustering of the high-risk behaviors and their association with job strain may be an interesting topic of future research.

Norito Kawakami (Department of Mental Health, University of Tokyo Graduate School of Medicine), Akizumi Tsutsumi (University of Occupational and Environmental Health), Takashi Haratani (Japan National Institute of Occupational Safety and Health), Fumio Kobayashi (Aichi Medical University), Masao Ishizaki (Kanazawa Medical University), Takeshi Hayashi (HITACHI Information & Telecommunication Systems Shinkawasaki Health Care Center), Osamu Fujita (Kariya General Hospital Eastern Division), Yoshiharu Aizawa (Kitasato University School of Medicine), Shogo Miyazaki (Meiji University Law School), Hisanori Hiro (Adecco Health Support Center), Takeshi Masumoto (Mitsubishi Chemical Corporation), Shuji Hashimoto (Fujita Health University School of Medicine), Shunichi Araki (National Institute of Occupational Safety and Health, Japan)

Corresponding author: Norito Kawakami, MD, Department of Mental Health, University of Tokyo Graduate School of Medicine, 7-3-1 Hongo, Bunkyo-ku, Tokyo 113-0033, Japan. (e-mail: norito@m.u-tokyo.ac.jp)
